# 2,10-Dihydr­oxy-13-methyl-13-aza­tetra­cyclo­[9.3.1.0^2,10^.0^3,8^]penta­deca-3(8),4,6-triene-9,15-dione

**DOI:** 10.1107/S1600536809015785

**Published:** 2009-05-07

**Authors:** J. Suresh, U. C. Nithya, R. Suresh Kumar, S. Perumal, P. L. Nilantha Lakshman

**Affiliations:** a Department of Physics, The Madura College, Madurai 625 011, India; b School of Chemistry, Madurai Kamaraj University, Madurai 625 021, India; c Department of Food Science and Technology, Faculty of Agriculture, University of Ruhuna, Mapalana, Kamburupitiya 81100, Sri Lanka

## Abstract

In the title compound, C_15_H_15_NO_4_, the *n*-methyl­piperidone ring adopts a chair conformation and both five-membered rings adopt a twist conformation. An intra­molecular O—H⋯O hydrogen bond is observed. Inversion-related mol­ecules are linked into *R*_2_^2^(10) dimers by pairs of O—H⋯O hydrogen bonds. The crystal structure is further stabilized by C—H⋯O hydrogen bonds.

## Related literature

For the biological activity of piperidine compounds, see: Watson *et al.* (2000 ▶). For ring conformation details, see: Cremer & Pople (1975 ▶).
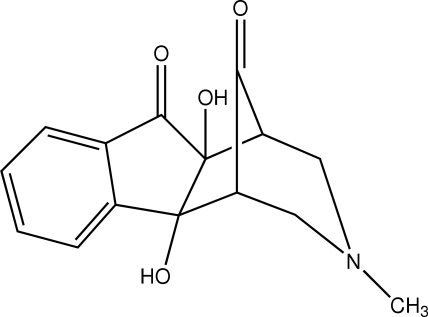


## Experimental

### 

#### Crystal data

C_15_H_15_NO_4_
*M* *_r_* = 273.28
Triclinic, 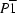

*a* = 7.5616 (7) Å
*b* = 8.9033 (8) Å
*c* = 10.8091 (11) Å
α = 72.764 (11)°
β = 80.486 (12)°
γ = 72.369 (11)°
*V* = 660.09 (11) Å^3^
*Z* = 2
Mo *K*α radiation
μ = 0.10 mm^−1^
*T* = 293 K
0.18 × 0.15 × 0.11 mm

#### Data collection

Nonius MACH-3 diffractometer
Absorption correction: ψ scan (North *et al.*, 1968 ▶) *T*_min_ = 0.982, *T*_max_ = 0.989
2890 measured reflections
2316 independent reflections
2025 reflections with *I* > 2σ(*I*)
*R*_int_ = 0.010
2 standard reflections frequency: 60 min intensity decay: none

#### Refinement

*R*[*F* ^2^ > 2σ(*F* ^2^)] = 0.037
*wR*(*F* ^2^) = 0.097
*S* = 1.11
2316 reflections
184 parameters
H-atom parameters constrained
Δρ_max_ = 0.22 e Å^−3^
Δρ_min_ = −0.23 e Å^−3^

### 

Data collection: *CAD-4 EXPRESS* (Enraf–Nonius, 1994 ▶); cell refinement: *CAD-4 EXPRESS*; data reduction: *XCAD4* (Harms & Wocadlo, 1996 ▶); program(s) used to solve structure: *SHELXS97* (Sheldrick, 2008 ▶); program(s) used to refine structure: *SHELXL97* (Sheldrick, 2008 ▶); molecular graphics: *PLATON* (Spek, 2009 ▶); software used to prepare material for publication: *SHELXL97*.

## Supplementary Material

Crystal structure: contains datablocks global, I. DOI: 10.1107/S1600536809015785/ci2789sup1.cif

Structure factors: contains datablocks I. DOI: 10.1107/S1600536809015785/ci2789Isup2.hkl

Additional supplementary materials:  crystallographic information; 3D view; checkCIF report

## Figures and Tables

**Table 1:** Hydrogen-bond geometry (Å, °)

*D*—H⋯*A*	*D*—H	H⋯*A*	*D*⋯*A*	*D*—H⋯*A*
O3—H3⋯O2	0.82	2.11	2.6080 (15)	119
O2—H2⋯O1^i^	0.82	1.90	2.7168 (15)	175
C2—H2*A*⋯O1^i^	0.98	2.59	3.3778 (18)	137
C18—H18*A*⋯O1^ii^	0.96	2.57	3.432 (2)	149
C5—H5⋯O3^iii^	0.98	2.46	3.4381 (18)	173

Symmetry codes: (i) 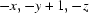; (ii) 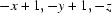; (iii) 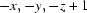.

**Fig. 1.**
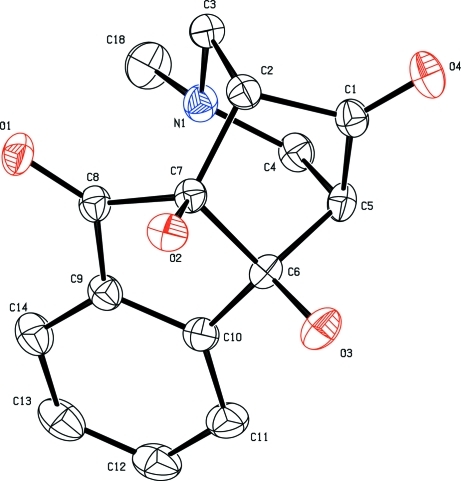
The molecular structure of the title compound, showing 30% probability displacement ellipsoids and the atom-numbering scheme. H atoms have been omitted for clarity.

**Fig. 2.**
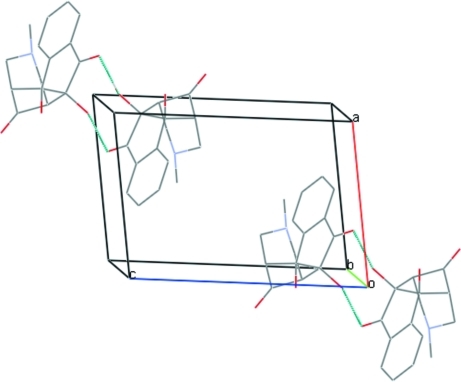
Partial packing view down the b axis. Hydrogen bonds are shown as dashed lines.

## supplementary crystallographic information

### Comment

The piperidine ring is a distinct structural feature of a variety of alkaloid natural products and drug candidates. Watson *et al.* (2000) observed that during the past decade there were thousands of piperidine compounds mentioned in clinical and preclinical studies. Piperidinones, though relatively less prominent, have also been regarded as precursors of a host of biologically active compounds and natural alkaloids, prior to their conversion to piperidines. Ninhydrin is a chemical used to detect ammonia or primary and secondary amines.

In the molecule of the title compound, (Fig. 1), the six-membered ring A (N1/C4/C5/C1/C2/C3), and the five membered rings B (C1/C2/C7/C6/C5) and C(C6-C10) are not planar. Rings B and C both adopt twist conformations, as indicated by Cremer & Pople (1975) puckering parameters Q = 0.455 (2) Å and Φ = 160.4 (2)° for ring B, and Q = 0.149 (2) Å and Φ = 21.6 (6)° for ring C. Ring A adopts a chair conformation.

In the crystal structure, the molecules are linked to form dimers by intermolecular O—H···O hydrogen bonds (Table 1), generating a graph set motif of *R*_2_^2^(10) (Fig.2). In addition, the structure is stabilized by C—H···O and van der Waals interactions.

### Experimental

A mixture of 1-methyl-4-piperidinone (0.2 g, 0.002 mol), ninhydrin (0.315 g, 0.002 mol) and sarcosine (0.156 g, 0.002 mol) in methanol (30 ml) was refluxed in a water bath for 10 h. After completion of the reaction as monitored by TLC, the excess solvent was removed under vacuum and the residue was subjected to flash column chromatography using petroleum ether-ethyl acetate mixture (8:2 *v*/*v*) as eluent to obtain crystals of title compound in 8% yield along with a other product (yield 13%, m.p. 447–448 K).

### Refinement

The H atoms were placed in calculated positions and allowed to ride on their carrier atoms, with C-H = 0.93–0.98 Å, O-H = 0.82 Å and *U*_iso_(H) = 1.2*U*_eq_(C) and 1.5*U*_eq_(C_methyl_,O).

### Crystal data

**Table:**

C_15_H_15_NO_4_	*Z* = 2
*M**_r_* = 273.28	*F*(000) = 288
Triclinic, *P*1	*D*_x_ = 1.375 Mg m^−^^3^
Hall symbol: -P 1	Mo *K*α radiation, λ = 0.71073 Å
*a* = 7.5616 (7) Å	Cell parameters from 25 reflections
*b* = 8.9033 (8) Å	θ = 2–25°
*c* = 10.8091 (11) Å	µ = 0.10 mm^−^^1^
α = 72.764 (11)°	*T* = 293 K
β = 80.486 (12)°	Block, colourless
γ = 72.369 (11)°	0.18 × 0.15 × 0.11 mm
*V* = 660.09 (11) Å^3^	

### Data collection

**Table:**

Nonius MACH-3 diffractometer	2025 reflections with *I* > 2σ(*I*)
Radiation source: fine-focus sealed tube	*R*_int_ = 0.010
graphite	θ_max_ = 25.0°, θ_min_ = 2.5°
ω–2θ scans	*h* = −1→8
Absorption correction: ψ scan (North *et al.*, 1968)	*k* = −10→10
*T*_min_ = 0.982, *T*_max_ = 0.989	*l* = −12→12
2890 measured reflections	2 standard reflections every 60 min
2316 independent reflections	intensity decay: none

### Refinement

**Table:**

Refinement on *F*^2^	Primary atom site location: structure-invariant direct methods
Least-squares matrix: full	Secondary atom site location: difference Fourier map
*R*[*F*^2^ > 2σ(*F*^2^)] = 0.037	Hydrogen site location: inferred from neighbouring sites
*wR*(*F*^2^) = 0.097	H-atom parameters constrained
*S* = 1.11	*w* = 1/[σ^2^(*F*_o_^2^) + (0.0454*P*)^2^ + 0.1839*P*] where *P* = (*F*_o_^2^ + 2*F*_c_^2^)/3
2316 reflections	(Δ/σ)_max_ = 0.001
184 parameters	Δρ_max_ = 0.22 e Å^−^^3^
0 restraints	Δρ_min_ = −0.23 e Å^−^^3^

### Special details

**Table:**

Geometry. All e.s.d.'s (except the e.s.d. in the dihedral angle between two l.s. planes) are estimated using the full covariance matrix. The cell e.s.d.'s are taken into account individually in the estimation of e.s.d.'s in distances, angles and torsion angles; correlations between e.s.d.'s in cell parameters are only used when they are defined by crystal symmetry. An approximate (isotropic) treatment of cell e.s.d.'s is used for estimating e.s.d.'s involving l.s. planes.
Refinement. Refinement of *F*^2^ against ALL reflections. The weighted *R*-factor *wR* and goodness of fit *S* are based on *F*^2^, conventional *R*-factors *R* are based on *F*, with *F* set to zero for negative *F*^2^. The threshold expression of *F*^2^ > σ(*F*^2^) is used only for calculating *R*-factors(gt) *etc*. and is not relevant to the choice of reflections for refinement. *R*-factors based on *F*^2^ are statistically about twice as large as those based on *F*, and *R*- factors based on ALL data will be even larger.

### Fractional atomic coordinates and isotropic or equivalent isotropic displacement parameters (Å^2^)

**Table:**

	*x*	*y*	*z*	*U*_iso_*/*U*_eq_	
O1	0.29809 (15)	0.41843 (13)	0.00498 (10)	0.0446 (3)	
O2	−0.04087 (14)	0.29519 (13)	0.08496 (10)	0.0421 (3)	
H2	−0.1139	0.3846	0.0581	0.063*	
O3	−0.02584 (16)	0.06248 (14)	0.30007 (11)	0.0499 (3)	
H3	−0.0724	0.0852	0.2313	0.075*	
N1	0.29935 (17)	0.38335 (15)	0.28995 (12)	0.0388 (3)	
O4	−0.19177 (16)	0.40313 (16)	0.45054 (11)	0.0538 (3)	
C8	0.2679 (2)	0.30815 (17)	0.09849 (13)	0.0353 (3)	
C4	0.2670 (2)	0.26334 (19)	0.41090 (14)	0.0403 (3)	
H4A	0.2445	0.3121	0.4832	0.048*	
H4B	0.3768	0.1712	0.4252	0.048*	
C10	0.3067 (2)	0.04870 (17)	0.24458 (14)	0.0372 (3)	
C7	0.07412 (19)	0.30768 (16)	0.17048 (13)	0.0322 (3)	
C5	0.0984 (2)	0.20371 (18)	0.40485 (13)	0.0375 (3)	
H5	0.0730	0.1229	0.4847	0.045*	
C6	0.1144 (2)	0.14491 (16)	0.28236 (13)	0.0348 (3)	
C9	0.3957 (2)	0.14516 (17)	0.14346 (14)	0.0372 (3)	
C1	−0.0622 (2)	0.35556 (19)	0.37706 (14)	0.0383 (3)	
C3	0.1375 (2)	0.52059 (18)	0.25385 (15)	0.0406 (3)	
H3A	0.1661	0.5959	0.1728	0.049*	
H3B	0.0969	0.5788	0.3209	0.049*	
C2	−0.0144 (2)	0.44610 (17)	0.23864 (13)	0.0362 (3)	
H2A	−0.1220	0.5275	0.1970	0.043*	
C14	0.5784 (2)	0.0851 (2)	0.09741 (16)	0.0486 (4)	
H14	0.6375	0.1503	0.0301	0.058*	
C11	0.3986 (2)	−0.11196 (19)	0.30063 (17)	0.0496 (4)	
H11	0.3394	−0.1777	0.3674	0.059*	
C12	0.5804 (3)	−0.1720 (2)	0.25488 (18)	0.0565 (5)	
H12	0.6442	−0.2794	0.2917	0.068*	
C13	0.6699 (2)	−0.0751 (2)	0.15496 (19)	0.0572 (5)	
H13	0.7927	−0.1183	0.1263	0.069*	
C18	0.4702 (3)	0.4293 (3)	0.2839 (2)	0.0624 (5)	
H18A	0.4881	0.5036	0.2010	0.094*	
H18B	0.5737	0.3334	0.2949	0.094*	
H18C	0.4615	0.4811	0.3517	0.094*	

### Atomic displacement parameters (Å^2^)

**Table:**

	*U*^11^	*U*^22^	*U*^33^	*U*^12^	*U*^13^	*U*^23^
O1	0.0438 (6)	0.0456 (6)	0.0332 (5)	−0.0098 (5)	0.0005 (4)	0.0021 (5)
O2	0.0419 (6)	0.0433 (6)	0.0416 (6)	−0.0060 (5)	−0.0146 (5)	−0.0116 (5)
O3	0.0522 (7)	0.0488 (6)	0.0518 (7)	−0.0269 (5)	−0.0081 (5)	−0.0022 (5)
N1	0.0360 (7)	0.0438 (7)	0.0393 (7)	−0.0155 (5)	0.0001 (5)	−0.0116 (5)
O4	0.0413 (6)	0.0706 (8)	0.0475 (7)	−0.0118 (6)	0.0104 (5)	−0.0234 (6)
C8	0.0365 (8)	0.0379 (7)	0.0285 (7)	−0.0084 (6)	−0.0037 (6)	−0.0053 (6)
C4	0.0413 (8)	0.0455 (8)	0.0329 (7)	−0.0085 (6)	−0.0075 (6)	−0.0095 (6)
C10	0.0422 (8)	0.0343 (7)	0.0341 (7)	−0.0068 (6)	−0.0073 (6)	−0.0090 (6)
C7	0.0320 (7)	0.0335 (7)	0.0289 (7)	−0.0067 (6)	−0.0057 (5)	−0.0053 (6)
C5	0.0396 (8)	0.0413 (8)	0.0268 (7)	−0.0130 (6)	0.0003 (6)	−0.0013 (6)
C6	0.0369 (8)	0.0322 (7)	0.0338 (7)	−0.0120 (6)	−0.0039 (6)	−0.0030 (6)
C9	0.0378 (8)	0.0387 (8)	0.0326 (7)	−0.0050 (6)	−0.0052 (6)	−0.0101 (6)
C1	0.0350 (8)	0.0468 (8)	0.0360 (8)	−0.0141 (6)	0.0002 (6)	−0.0135 (6)
C3	0.0491 (9)	0.0361 (8)	0.0370 (8)	−0.0128 (7)	−0.0008 (6)	−0.0100 (6)
C2	0.0349 (7)	0.0354 (7)	0.0339 (7)	−0.0028 (6)	−0.0053 (6)	−0.0080 (6)
C14	0.0396 (8)	0.0557 (10)	0.0453 (9)	−0.0037 (7)	−0.0015 (7)	−0.0160 (8)
C11	0.0600 (10)	0.0342 (8)	0.0482 (9)	−0.0044 (7)	−0.0111 (8)	−0.0064 (7)
C12	0.0617 (11)	0.0404 (9)	0.0592 (11)	0.0085 (8)	−0.0191 (9)	−0.0161 (8)
C13	0.0442 (9)	0.0592 (11)	0.0625 (11)	0.0096 (8)	−0.0102 (8)	−0.0286 (9)
C18	0.0456 (10)	0.0723 (12)	0.0787 (13)	−0.0281 (9)	0.0013 (9)	−0.0244 (10)

### Geometric parameters (Å, °)

**Table:**

O1—C8	1.2285 (17)	C5—C1	1.509 (2)
O2—C7	1.4171 (16)	C5—C6	1.537 (2)
O2—H2	0.82	C5—H5	0.98
O3—C6	1.4219 (17)	C9—C14	1.387 (2)
O3—H3	0.82	C1—C2	1.517 (2)
N1—C3	1.453 (2)	C3—C2	1.541 (2)
N1—C18	1.455 (2)	C3—H3A	0.97
N1—C4	1.4613 (19)	C3—H3B	0.97
O4—C1	1.2088 (18)	C2—H2A	0.98
C8—C9	1.471 (2)	C14—C13	1.385 (3)
C8—C7	1.5419 (19)	C14—H14	0.93
C4—C5	1.539 (2)	C11—C12	1.381 (3)
C4—H4A	0.97	C11—H11	0.93
C4—H4B	0.97	C12—C13	1.388 (3)
C10—C11	1.386 (2)	C12—H12	0.93
C10—C9	1.391 (2)	C13—H13	0.93
C10—C6	1.507 (2)	C18—H18A	0.96
C7—C2	1.5443 (19)	C18—H18B	0.96
C7—C6	1.5720 (18)	C18—H18C	0.96
			
C7—O2—H2	109.5	C14—C9—C8	128.62 (14)
C6—O3—H3	109.5	C10—C9—C8	109.95 (13)
C3—N1—C18	114.32 (13)	O4—C1—C5	128.54 (14)
C3—N1—C4	113.97 (12)	O4—C1—C2	126.94 (14)
C18—N1—C4	112.86 (13)	C5—C1—C2	104.23 (11)
O1—C8—C9	126.53 (13)	N1—C3—C2	105.67 (11)
O1—C8—C7	124.00 (12)	N1—C3—H3A	110.6
C9—C8—C7	108.44 (12)	C2—C3—H3A	110.6
N1—C4—C5	110.50 (11)	N1—C3—H3B	110.6
N1—C4—H4A	109.6	C2—C3—H3B	110.6
C5—C4—H4A	109.6	H3A—C3—H3B	108.7
N1—C4—H4B	109.6	C1—C2—C3	103.05 (11)
C5—C4—H4B	109.6	C1—C2—C7	103.33 (11)
H4A—C4—H4B	108.1	C3—C2—C7	109.41 (11)
C11—C10—C9	120.35 (15)	C1—C2—H2A	113.4
C11—C10—C6	128.39 (14)	C3—C2—H2A	113.4
C9—C10—C6	111.20 (12)	C7—C2—H2A	113.4
O2—C7—C8	108.50 (11)	C13—C14—C9	117.82 (16)
O2—C7—C2	114.39 (11)	C13—C14—H14	121.1
C8—C7—C2	115.40 (12)	C9—C14—H14	121.1
O2—C7—C6	108.69 (11)	C12—C11—C10	118.30 (16)
C8—C7—C6	103.21 (11)	C12—C11—H11	120.8
C2—C7—C6	105.84 (11)	C10—C11—H11	120.8
C1—C5—C6	100.91 (11)	C11—C12—C13	121.32 (15)
C1—C5—C4	105.49 (12)	C11—C12—H12	119.3
C6—C5—C4	112.08 (11)	C13—C12—H12	119.3
C1—C5—H5	112.5	C14—C13—C12	120.77 (16)
C6—C5—H5	112.5	C14—C13—H13	119.6
C4—C5—H5	112.5	C12—C13—H13	119.6
O3—C6—C10	114.30 (12)	N1—C18—H18A	109.5
O3—C6—C5	106.98 (11)	N1—C18—H18B	109.5
C10—C6—C5	114.94 (12)	H18A—C18—H18B	109.5
O3—C6—C7	111.58 (11)	N1—C18—H18C	109.5
C10—C6—C7	104.97 (11)	H18A—C18—H18C	109.5
C5—C6—C7	103.62 (11)	H18B—C18—H18C	109.5
C14—C9—C10	121.43 (14)		
			
C3—N1—C4—C5	54.45 (16)	C6—C10—C9—C14	176.26 (14)
C18—N1—C4—C5	−172.98 (13)	C11—C10—C9—C8	178.38 (14)
O1—C8—C7—O2	66.57 (17)	C6—C10—C9—C8	−4.31 (17)
C9—C8—C7—O2	−102.49 (12)	O1—C8—C9—C14	4.9 (3)
O1—C8—C7—C2	−63.28 (18)	C7—C8—C9—C14	173.60 (15)
C9—C8—C7—C2	127.66 (12)	O1—C8—C9—C10	−174.49 (14)
O1—C8—C7—C6	−178.24 (13)	C7—C8—C9—C10	−5.78 (16)
C9—C8—C7—C6	12.70 (14)	C6—C5—C1—O4	138.05 (16)
N1—C4—C5—C1	−56.97 (15)	C4—C5—C1—O4	−105.16 (17)
N1—C4—C5—C6	51.96 (16)	C6—C5—C1—C2	−47.81 (13)
C11—C10—C6—O3	−48.1 (2)	C4—C5—C1—C2	68.98 (13)
C9—C10—C6—O3	134.84 (13)	C18—N1—C3—C2	168.31 (13)
C11—C10—C6—C5	76.15 (19)	C4—N1—C3—C2	−59.82 (15)
C9—C10—C6—C5	−100.88 (14)	O4—C1—C2—C3	98.33 (17)
C11—C10—C6—C7	−170.70 (15)	C5—C1—C2—C3	−75.95 (13)
C9—C10—C6—C7	12.27 (15)	O4—C1—C2—C7	−147.76 (15)
C1—C5—C6—O3	−80.03 (13)	C5—C1—C2—C7	37.97 (14)
C4—C5—C6—O3	168.14 (11)	N1—C3—C2—C1	69.03 (14)
C1—C5—C6—C10	151.91 (12)	N1—C3—C2—C7	−40.39 (14)
C4—C5—C6—C10	40.09 (16)	O2—C7—C2—C1	106.41 (13)
C1—C5—C6—C7	37.97 (13)	C8—C7—C2—C1	−126.65 (12)
C4—C5—C6—C7	−73.85 (14)	C6—C7—C2—C1	−13.22 (14)
O2—C7—C6—O3	−23.90 (15)	O2—C7—C2—C3	−144.35 (12)
C8—C7—C6—O3	−138.96 (12)	C8—C7—C2—C3	−17.42 (15)
C2—C7—C6—O3	99.41 (13)	C6—C7—C2—C3	96.02 (13)
O2—C7—C6—C10	100.42 (12)	C10—C9—C14—C13	0.3 (2)
C8—C7—C6—C10	−14.64 (14)	C8—C9—C14—C13	−178.97 (15)
C2—C7—C6—C10	−136.28 (11)	C9—C10—C11—C12	1.0 (2)
O2—C7—C6—C5	−138.66 (11)	C6—C10—C11—C12	−175.81 (15)
C8—C7—C6—C5	106.28 (12)	C10—C11—C12—C13	−0.3 (3)
C2—C7—C6—C5	−15.36 (14)	C9—C14—C13—C12	0.4 (3)
C11—C10—C9—C14	−1.1 (2)	C11—C12—C13—C14	−0.4 (3)

### Hydrogen-bond geometry (Å, °)

**Table:**

*D*—H···*A*	*D*—H	H···*A*	*D*···*A*	*D*—H···*A*
O3—H3···O2	0.82	2.11	2.6080 (15)	119
O2—H2···O1^i^	0.82	1.90	2.7168 (15)	175
C2—H2A···O1^i^	0.98	2.59	3.3778 (18)	137
C18—H18A···O1^ii^	0.96	2.57	3.432 (2)	149
C5—H5···O3^iii^	0.98	2.46	3.4381 (18)	173

Symmetry codes: (i) −*x*, −*y*+1, −*z*; (ii) −*x*+1, −*y*+1, −*z*; (iii) −*x*, −*y*, −*z*+1.
